# Role of Convex Probe Endobronchial Ultrasound in the Diagnosis and Treatment of Nonmalignant Diseases

**DOI:** 10.1155/2019/6838439

**Published:** 2019-06-17

**Authors:** Ahmed A. Aljohaney

**Affiliations:** Department of Medicine, King Abdulaziz University, Jeddah, Saudi Arabia

## Abstract

Here we present a comprehensive review of the literature concerning the utility of convex probe endobronchial ultrasound (CP-EBUS) in the diagnosis and treatment of nonmalignant conditions and discuss the associated complications. CP-EBUS has been conventionally used for the staging of lung cancer and sampling of mediastinal and hilar nodes. However, its application is not limited to malignant conditions, and it is gaining acceptance as a diagnostic modality of choice for nonmalignant conditions such as tuberculosis, sarcoidosis, pulmonary embolism, thyroid lesions, and cysts. Moreover, its therapeutic value allows for extended applications such as mediastinal and thyroid cyst drainage, fiducial marker placement for radiation therapy, and transbronchial needle injection. The noninvasiveness, low complication rate, high diagnostic yield, and satisfactory sensitivity and specificity values are the main attributes that lend credence to the use of CP-EBUS as a standalone primary diagnostic and therapeutic tool in pulmonary medicine in the foreseeable future.

## 1. Introduction

**Figure 1:**
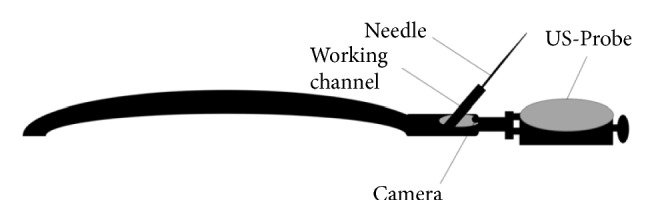
Schematic presentation of features of CP-EBUS equipped with video camera and needle for sampling and a convex ultrasound probe.

In 1897, a metal tube with an electric light served as the first rigid bronchoscope for the removal of pork bone from the airway [1]. This preliminary design was a breakthrough in the history of pulmonary medicine. In 1904, rigid bronchoscopy crossed its first milestone when the devices were equipped with a suction channel and small light bulb for illumination. In 1966, Ikeda designed the first flexible bronchoscope; however, the first commercially available flexible bronchoscope was introduced by Machita and Olympus in 1968. Video bronchoscopy became available for educational purposes in 1987, following which it evolved and came to be used by surgeons for the monitoring of real-time images of procedures on computer screens [2]. Continuous developments and improvements resulted in the invention of endobronchial ultrasound (EBUS) in the early 1990s. At that time, a radial probe, which provided better resolution and superior image quality, was used. Although radial probe EBUS (RP-EBUS) provides images of the deeper parts of the airway, it does not facilitate real-time transbronchial needle aspiration (TBNA). This limitation was overcome by the development of convex probe EBUS (CP-EBUS) [3], following which there have been few improvements in the appearance of bronchoscopes.  Table 1 presents a comparison of RP-EBUS and CP-EBUS, and Figure 1 presents the schematic features of CP-EBUS.

Because of its noninvasiveness, CP-EBUS-guided real-time TBNA has largely replaced invasive sampling and diagnostic modalities such as mediastinoscopy. The value of CP-EBUS for the diagnosis and staging of lung cancer is well known. CP-EBUS can also aid in the diagnosis of nonmalignant conditions such as tuberculosis, sarcoidosis, and pulmonary embolism and facilitate treatments such as bronchogenic cyst drainage and transbronchial injection [3].

Here we present a comprehensive review of the literature regarding the utility of CP-EBUS for nonmalignant conditions, with focus on its expanded prospects in terms of diagnosis and treatment. Complications associated with CP-EBUS are also discussed.

## 2. Main Text

### 2.1. Diagnostic Value of CP-EBUS

#### 2.1.1. Pulmonary Sarcoidosis

Sarcoidosis is a multisystemic inflammatory disorder with an unknown etiology that is characterized by the accumulation of macrophages and the formation of epithelioid cell nonnecrotizing benign granulomas. The lungs and hilar or mediastinal nodes are affected in 90% patients with sarcoidosis; therefore, pulmonary sarcoidosis is the most common form of the disease. Diagnostic modalities include computed tomography (CT), transbronchial lung biopsy (TBLB), endobronchial biopsy (EBB), TBNA, and mediastinoscopy. Pneumothorax and haemoptysis are major side effects of TBLB, which also exhibits moderate diagnostic sensitivity. Although mediastinoscopy is highly effective in the diagnosis of sarcoidosis, the associated costs and invasiveness limit its routine use. CP-EBUS-guided TBNA is an effective, noninvasive approach for sampling on an outpatient basis. Wong* et al.* and Garwood* et al.* first reported the use of CP-EBUS-guided TBNA for the diagnosis of sarcoidosis, with diagnostic sensitivities of 91.8% and 85%, respectively [4, 5]. In another prospective study of 39 patients, EBUS-TBNA showed a sensitivity, specificity, positive predictive value, and diagnostic accuracy of 93.9%, 100%, 100%, and 94.8%, respectively [6]. A meta-analysis of 14 studies involving 2097 patients who underwent EBUS-TBNA for the diagnosis of sarcoidosis reported a pooled diagnostic yield of 0.79, pooled sensitivity of 0.84, and pooled specificity of 1.00 [7]. CP-EBUS is also used to study the echogenic features of lymph nodes in patients with sarcoidosis. A retrospective analysis found homogeneous low echogenicity and the presence of a germinal central structure as the main characteristics of lymph nodes in patients with sarcoidosis [8]. The presence of granules in lymph nodes reportedly exhibits a diagnostic accuracy of 99.3% [9]. Echogenicity analysis using EBUS-TBNA also aids in the differentiation of sarcoidosis from malignancy, as well as the differentiation of benign lymphadenopathies, including sarcoidosis, from cancer recurrence [10].

Several studies have compared sensitivity and accuracy values among various diagnostic modalities for sarcoidosis. Oki* et al.* presented a positive pathological diagnosis rate of 93% for EBUS-TBNA or TBNA alone and 100% for EBUS-TBNA combined with TBNA [11]. In another study, the diagnostic sensitivity of EBUS-TBNA was significantly higher (85%) than that of standard bronchoscopy techniques (35%) [12]. Comparisons of EBUS-TBNA with TBLB have been presented with contradictory results. A prospective study of 62 patients reported a significantly higher diagnostic yield for EBUS-TBNA (94%) than for TBLB (37%) [13], whereas another study found no significant difference in the diagnostic accuracy of both techniques [14]. In another cohort of 33 patients with sarcoidosis, the diagnostic sensitivities of EBUS-TBNA, TBLB, EBB, and bronchoalveolar lavage (BAL) were 90%, 35%, 6%, and 71%, respectively [15]. However, in a randomized controlled trial including 130 patients, EBUS-TBNA showed a higher diagnostic yield (74.5%) than did TBNA (48.4%) and EBB (36.3%), with no significant difference from TBLB (69.6%). A meta-analysis of studies including 1823 patients with sarcoidosis compared the diagnostic yield of EBUS-TBNA with that of TBLB and found odds ratios of 0.26 and 126.58, respectively [16]. Finally, EUS-FNA and transbronchial lung cryobiopsy showed no significant difference in the diagnostic yield (66.7%), with a combination of the two modalities increasing the diagnostic yield to 100% [17].

Multiple factors influencing the sensitivity of EBUS-TBNA have been studied; these include the gauge size, lymph node size, disease stage, number of sampled lymph node stations, and number of needle passes per lymph node. A prospective, randomized, double-blind trial found no significant difference between 21G and 22G needles in terms of the diagnostic yield of EBUS-TBNA in patients with sarcoidosis [18]. However, the yield of 19G eXcelon core needles was superior to that of 21G EBUS needles in a study of EBUS-TBNA from mediastinal lymph nodes in patients with sarcoidosis; this was attributed to the tissue size obtained with the former needle [9]. In a retrospective chart review of EBUS-TBNA performed with a 19G needle, the diagnostic yield of EBUS-TBNA was significantly higher (93.3%) than that of TBLB (38%) and EBB (43%) [19]. The diagnostic sensitivity of EBUS-TBNA has also been associated with the lymph node size [20] and disease stage [21].

In summary, direct comparisons between conventional diagnostic modalities and EBUS-TBNA have favored the use of EBUS-TBNA as a primary diagnostic tool for sarcoidosis [22].

#### 2.1.2. Tuberculosis

Infectious manifestation of* Mycobacterium tuberculosis* is a global public health concern for clinicians and researchers. Sputum or smear culture remains the gold standard for the diagnosis of pulmonary tuberculosis (TB). However, these methods are not efficient for the diagnosis of mediastinal tuberculous lymphadenopathy (TBLA). Moreover, 25% and 60% patients have negative sputum smears and no spontaneous sputum production, respectively [23]. EBUS-TBNA is a noninvasive modality that has been found to aid in the diagnosis of pulmonary TB and TBLA. It helps in obtaining adequate lymphocytic material to test for acid-fast bacilli by staining and examine for the presence of necrotizing granulomas. Steinfort* et al.* first used EBUS-TBNA for the diagnosis of mediastinal intrathoracic lymphadenopathy in patients infected with the human immunodeficiency virus (HIV) [24]. Subsequently, Lyu* et al*. demonstrated the use of EBUS-TBNA for the diagnosis of multidrug-resistant TB [25]. While one retrospective study assessing the diagnosis of TBLA reported a diagnostic yield of 64.6% for EBUS-TBNA [26], another prospective study of patients with intrathoracic TB reported a diagnostic accuracy of 90%, sensitivity of 85%, and specificity of 100% for the same modality [27]. Similar diagnostic specificity (100%) and sensitivity (90.9%) values were shown in a cohort of 93 patients [28]. A meta-analysis of studies involving 809 patients presented pooled sensitivity and specificity values of 0.80 and 1.00, respectively, for the diagnosis of intrathoracic TB by EBUS-TBNA. However, the pooled sensitivity for the diagnosis of intrathoracic TBLA was higher at 0.87. In another meta-analysis of studies involving 809 patients, the pooled diagnostic yield of EBUS-TBNA for TBLA was 80% [29]. EBUS-TBNA was also found to exhibit a diagnostic yield as high as 96.6% in cases of mediastinal TB, which presents a diagnostic challenge for clinicians [30].

#### 2.1.3. Fungal Infection

Conventionally, diagnosis of fungal infection-related lymphadenopathy requires tissue biopsy for obtaining adequate material for fungal culture. However, Sodhi* et al*. confirmed the utility of EBUS-TBNA for the diagnosis of histoplasmosis in 452 patients, with a diagnostic yield of 78% [31]. In another study, EBUS helped in confirming the diagnosis of histoplasmosis, with no requirement for further invasive procedures [32]. EBUS was also found to be effective in the diagnosis of pulmonary mucormycosis in a debilitated patient who was not fit enough to undergo invasive procedures [33]. The use of EBUS also facilitates rapid diagnosis in patients with other endemic fungal infections such as coccidioidomycosis [34].

#### 2.1.4. Reactive Hyperplasia

Reactive hyperplasia involving the mediastinal and hilar lymph nodes is a nonspecific diagnosis. The condition is potentially caused by benign etiologies such as fungal infection [32]. CP-EBUS can reliably provide adequate material for the diagnosis of reactive hyperplasia with a high degree of confidence. In one study, operators diagnosed reactive hyperplasia using CP-EBUS in 60% patients [35]. Furthermore, Ko* et al*. confirmed the accuracy of CP-EBUS in diagnosing reactive hyperplasia in a cohort of patients suspected to have lymphoma or other nonneoplastic lesions [36].

#### 2.1.5. Nocardiosis

Nocardiosis is an infectious disease caused by* Nocardia* spp. Fujikura* et al.* successfully used CP-EBUS to sample purulent exudates containing* N. asteroids* [37]. CP-EBUS-guided TBNA was also used to isolate two* Nocardia* strains from a lung mass in a patient who had undergone kidney–pancreas transplantation [38]. These findings indicate the diagnostic value of CP-EBUS for nonmalignant mediastinal lymphadenopathy.

#### 2.1.6. Pulmonary Thromboembolism

Pulmonary embolism (PE) occurs when a blood clot gets lodged in blood vessels anywhere in the lungs, consequently blocking blood flow to the relevant parts of the lungs. It is a common disease and can be life-threatening depending on the presence of comorbid conditions, anatomical site, and size of the clot. Thus, prompt diagnosis is essential. Contrast-enhanced CT, ventilation perfusion scanning, and, less commonly, pulmonary arteriography are the preferred diagnostic methods. However, these techniques cannot be used for individuals with contraindications for the use of contrast agents, such as allergy or renal failure, pregnancy, and hemodynamic instability. In such cases, CP- EBUS serves as an effective alternative diagnostic modality.

**Figure 2:**
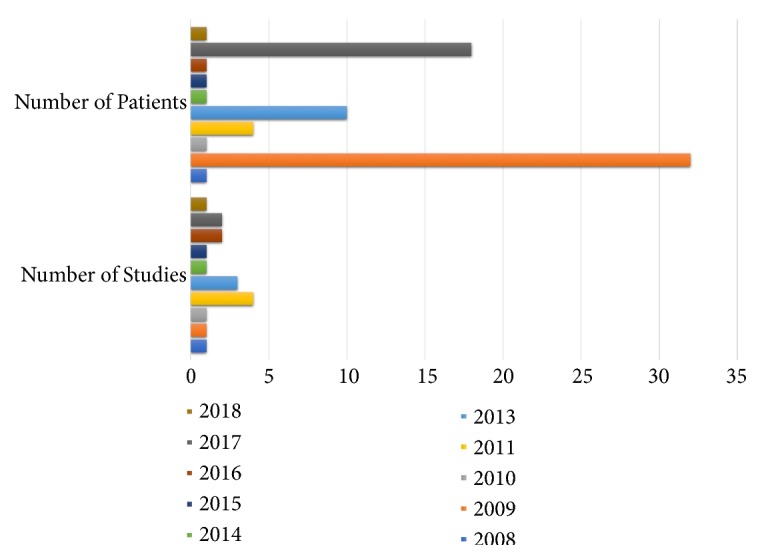
Number of studies and patients per year diagnosed with Pulmonary Embolism through CP-EBUS.

The pulmonary arteries are located in close proximity to the bronchial and tracheal airways and can be easily visualized by CP-EBUS. CP-EBUS equipped with color Doppler imaging enables the pulmonologist to characterize the size of the thrombus and the extent of the blockage in real time. Casoni* et al*. initially reported the use of EBUS-CP for the diagnosis of PE in a 26-year-old patient [39]. Subsequently, Aumiller* et al*. conducted a multicenter pilot study involving the use of CP-EBUS for the diagnosis of PE after angio-CT in 32 patients. Angio-CT detected 101 PEs while CP-EBUS detected only 97. However, CP-EBUS diagnosed at least one thrombus per patient, which was sufficient to establish the diagnosis. Three thrombi in the left upper lobe and one in the middle lobe could not be detected by CP-EBUS. The duration of CP-EBUS was 5 min/patient for the first 16 patients and 3 min/patient thereafter [40]. In 2010, CP-EBUS attempted for TBNA identified PE in the right pulmonary artery in a 69-year-old man. PE was then confirmed by angio-CT [41]. Subsequently, four independent case studies were published in 2011, with each describing a single patient aged 61–83 years who was diagnosed with PE by CP-EBUS [42–46]. The high sensitivity of CP-EBUS was further highlighted by Santaolalla, who presented an unusual case where EBUS-TBNA detected PE after angio-CT failed to do so [44]. Another study involving eight patients monitored for PE in 2013 exhibited encouraging outcomes. The authors indicated the feasibility of EBUS for sampling and diagnosis. When 548 CP-EBUS procedures were performed in one study, four cases of PE, three of which also involved cancer, were detected [47]. In yet another study, 14 patients were diagnosed with PE using EBUS-TBNA [39].  Figure 2 presents the number of studies and patients diagnosed with PE using CP-EBUS on an annual basis.

CP-EBUS can easily visualize and monitor the aortic arch, left and right pulmonary artery trunks, azygos vein, hilum, and lobar arteries; however, the image quality is generally suboptimal because of the low frequency (5–10MHz). Consequently, confirmation by angio-CT is required. Furthermore, the probe diameter of 6.3–6.9 mm facilitates the visualization of clots only in adjacent vascular structures. Nevertheless, CP-EBUS is a safe and sensitive method for real-time evaluation of PE, enabling not only diagnosis but also real-time evaluation of thrombolytic therapies in clinical studies [3].

#### 2.1.7. Thyroid Cysts, Lesions, and Nodules

EBUS-TBNA has been infrequently used for thyroid aspiration. Chalhoub e*t al.* initially reported the use of EBUS-TBNA for the diagnosis of substernal solitary thyroid nodules [48]. In another study, EBUS-TBNA was performed when ultrasound characteristics suggested a cyst. A sample was obtained for histopathological analysis, which revealed plaques and clusters of follicular cells, abundant macrophages, and hemosiderophages. Thus, the diagnosis of a thyroid cyst was confirmed [49]. Casal* et al*. reported 12 cases of thyroid biopsy performed using EBUS-TBNA [50]. More recently, Ozturk* et al.* reported three cases of mediastinal ectopic thyroid diagnosed by EBUS-TBNA [51].

#### 2.1.8. Nonthrombotic Endovascular Lesions (NELs)

Al-Saffar* et al.* systematically reviewed 12 cases where EBUS-TBNA was used for the diagnosis of NELs in the pulmonary artery and lungs. The diagnoses included sarcoma (*n *= 6), lung cancer (*n *= 2), thyroid cancer (*n *= 1), renal cell cancer (*n *= 1), melanoma (*n *= 1), and PE (*n *= 1) [52]. The findings indicated that EBUS-TBNA is a safe and efficient diagnostic modality for NELs and tumor embolisms.

### 2.2. Therapeutic Value of CP-EBUS

#### 2.2.1. Fiducial Marker Placement

CP-EBUS is reportedly used for the placement of fiducial markers for tracking purposes in patients requiring stereotactic radiation therapy and CyberKnife therapy for mediastinal and lung cancers [53, 54]. Harley* et al.* successfully used CP-EBUS for the placement of 2–5 fiducial markers around tumor masses in 43 patients scheduled for radiosurgery [55].

#### 2.2.2. Transbronchial Needle Injection (TBNI)

EBUS-TBNI in patients with mediastinal diseases is another therapeutic application of CP-EBUS. Two independent case studies have documented EBUS-TBNI of cisplatin for the treatment of lung cancer [56, 57]. In addition, Parikh* et al.* used EBUS-TBNI of liposomal amphotericin B for the treatment of symptomatic aspergilloma [58].

#### 2.2.3. Drainage

Nakajima* et al. *initially reported the therapeutic use of EBUS-TBNA for aspiration during the treatment of central airway stenosis caused by a mediastinal cyst. The Doppler mode in CP-EBUS helps in the differentiation of cysts from vascular structures [59]. Drainage of infectious bronchogenic cysts using EBUS-TBNA, with subsequent resolution of symptoms, has also been reported [60, 61]. As mentioned above, EBUS-TBNA is also used in the diagnosis and treatment of thyroid cysts [62].

### 2.3. Complications and Limitations

CP-EBUS is a noninvasive procedure with an excellent safety profile. Although it has several applications beyond the diagnosis of malignancies, it cannot cover the entire mediastinum and allows visualization of only the anterosuperior portion. Moreover, some anatomical locations such as the upper lobes cannot be accessed by CP-EBUS [63]. A nationwide survey by the Japan Society for Respiratory Endoscopy reported a complication rate of only 1.23% for 7,345 cases in 210 facilities. Hemorrhage was the most frequent complication (*n *= 50), followed by infectious complications (*n *= 14), breakage of the ultrasound bronchoscope (*n* = 98), and needle puncture (*n* = 15) [64].

## 3. Conclusions

In summary, CP-EBUS is a minimally invasive procedure that plays a pivotal role in the diagnosis and treatment of malignant and nonmalignant mediastinal diseases. CP-EBUS-guided TBNA provides the highest diagnostic yield in cases involving nonmalignant mediastinal diseases such as tuberculosis and sarcoidosis. However, combination with TBLB and EBB increases the yield to 100%, with minimal complications. Furthermore, CP-EBUS can provide adequate information for the diagnosis of most benign diseases without the need for other invasive procedures such as mediastinoscopy. There is overwhelming evidence on the efficacy and safety of CP-EBUS for benign conditions, with the noninvasiveness, low complication rate, high diagnostic yield, and satisfactory sensitivity and specificity values comprising the main attributes that lend credence to its use as a standalone primary diagnostic and therapeutic tool in pulmonary medicine. Therefore, the acquisition of this technology is highly recommended for most institutions to enable pulmonologists to safely and effectively diagnose benign and malignant diseases of the mediastinum.

## Figures and Tables

**Table 1:** Comparison of RP-EBUS and CP-CBUS.

	CP-EBUS	RP-EBUS
Flexibility of bronchoscope for view	60°	360°
Real time TBNA Possibility	Yes	No
Doppler mode	Yes	No
Frequency of Ultrasound probe**∗**	5-12MHz	20MHz
Tissue Penetration	Low	High

*∗*A higher frequency leads to better resolution and image quality and less penetration and a low frequency in ultrasound waves leads to better penetration yet low quality image due to low resolution.
